# Repurposing the anti-epileptic drug sodium valproate as an adjuvant treatment for diffuse intrinsic pontine glioma

**DOI:** 10.1371/journal.pone.0176855

**Published:** 2017-05-25

**Authors:** Clare L. Killick-Cole, William G. B. Singleton, Alison S. Bienemann, Daniel J. Asby, Marcella J. Wyatt, Lisa J. Boulter, Neil U. Barua, Steven S. Gill

**Affiliations:** 1 Functional Neurosurgery Research Group, School of Clinical Sciences, University of Bristol, Learning & Research Building, Southmead Hospital, Bristol, United Kingdom; 2 Department of Neurosurgery, North Bristol NHS Trust, Bristol, United Kingdom; Universidad de Navarra, SPAIN

## Abstract

Targeting epigenetic changes in diffuse intrinsic pontine glioma (DIPG) may provide a novel treatment option for patients. This report demonstrates that sodium valproate, a histone deacetylase inhibitor (HDACi), can increase the cytotoxicity of carboplatin in an additive and synergistic manner in DIPG cells *in vitro*. Sodium valproate causes a dose-dependent decrease in DIPG cell viability in three independent *ex vivo* cell lines. Furthermore, sodium valproate caused an increase in acetylation of histone H3. Changes in cell viability were consistent with an induction of apoptosis in DIPG cells *in vitro*, determined by flow cytometric analysis of Annexin V staining and assessment of apoptotic markers by western blotting. Subsequently, immunofluorescent staining of neuronal and glial markers was used to determine toxicity in normal rat hippocampal cells. Pre-treatment of cells with sodium valproate enhanced the cytotoxic effects of carboplatin, in three DIPG cell lines tested. These results demonstrate that sodium valproate causes increased histone H3 acetylation indicative of HDAC inhibition, which is inversely correlated with a reduction in cell viability. Cell viability is reduced through an induction of apoptosis in DIPG cells. Sodium valproate potentiates carboplatin cytotoxicity and prompts further work to define the mechanism responsible for the synergy between these two drugs and determine *in vivo* efficacy. These findings support the use of sodium valproate as an adjuvant treatment for DIPG.

## Introduction

Whilst great advances have been made in the characterisation of the molecular changes in DIPG, the clinical challenges that oppose treatment strategies remain set in place. Previous clinical trials have been reliant on the assumption of similar genetics between adult high grade glioma and DIPG, however, recent discoveries of *H3F3A* and *AVCR1* mutations have led to the emergence of several preclinical studies with DIPG targeted therapies [1–6]. The current DIPG treatment regime consists of radiotherapy that provides only a palliative response for patients and it is well known that cranial radiation alone in children can cause neurological deficits, further prompting the urgent need for the development of more efficacious and less toxic treatment plans for these patients. Presently, median survival for DIPG patients is nine months with mortality rates of 90% by 18 months from diagnosis [7, 8]. These statistics authenticate the clear unmet clinical need for DIPG patients and the requirement for readily translatable treatments. This study examines the *in vitro* effect of pre-sensitising DIPG cells by epigenetic regulation with sodium valproate to enhance the cytotoxic effects of carboplatin.

One of the most frequent mutations occurring in DIPG arises in the *H3F3A* gene, which encodes histone H3.3 [9, 10]. Other histone mutations occur in a mutually exclusive manner, with alterations of *HIST1H3B* modifying histone H3.1. These mutations are considered the driving force of tumorigenesis by reducing histone K27 methylation, which results in gene expression alterations in cells of the developing brain stem [11].

Sodium valproate has been used clinically for a number of years and is a well-established drug for the long-term treatment of epilepsy. More recently, it was proven to be a HDAC inhibitor (HDACi) and exerts anti-tumour activity towards several different cancer types *in vitro [12–14]*. Currently, epigenetic therapies are at the forefront of cancer research, with HDACi in particular being approved for use against haematological malignancies, where it has been shown to induce differentiation and apoptosis of leukaemia cells *in vitro* and in clinical trials *[15]*. Providing a rationale for this study are the recent publications purporting the efficacy of HDACi for the treatment of DIPG [16, 17]. To enable the rapid translation of already approved drugs, we have identified sodium valproate as a potential adjuvant chemotherapeutic against DIPG, owing to its HDAC inhibition, anti-cancer activity demonstrated in several tumour types *in vitro* and *in vivo* [12, 18, 19] and a well-known toxicity profile. Furthermore, its ability to cross the blood brain barrier (BBB) favours its use as a treatment for brain malignancies. We hypothesised that sodium valproate would not only cause cytotoxicity to DIPG cells as a monotherapy, but that its HDAC inhibition would sensitise cells to DNA intercalating chemotherapeutics, such as carboplatin. As such, our research focuses on establishing *in vitro* preclinical evidence to support the use of sodium valproate for the treatment of DIPG.

Monotherapies using sodium valproate have shown limited success in clinical trials, with only 5% of acute myelogenous leukaemia patients showing response to sodium valproate treatment [15]. Given the mechanism of action, it is viable that sodium valproate treatment may alter how DIPG cells respond to other chemotherapeutics, such as DNA intercalating agents. Such combinations have been used in medulloblastoma and glioma studies, whereby sodium valproate treatment was combined with topoisomerase inhibitors and enhanced the cytotoxicity of topotecan and etoposide [20, 21].

The advent of convection enhanced delivery (CED) has provided a direct route for drug administration to the brain, circumventing problems associated with drugs crossing the blood brain barrier (BBB) [22]. CED has allowed discrete areas of the brain to be precisely targeted for the delivery of treatments to brain tumours. Intermittent CED of carboplatin has been used by our group to treat patients with DIPG and glioblastoma, utilising an implanted drug delivery system that allows repeated infusions without the need for repeated surgery [23, 24].

Epigenetics are now of great importance in cancer biology, with the regulation of the epigenome by both HDAC and DNA Methyltransferase inhibitors being of keen interest. Here, we present findings that demonstrate sodium valproate, a commonly used anti-epileptic drug, as a potential adjuvant and neoadjuvant chemotherapeutic, especially when combined with carboplatin, a drug recently used by CED for DIPG patients [23, 25]. These results support the rationale for further investigation into the clinical efficacy of sodium valproate as a cancer therapeutic.

## Methods

### Cell culture

Patient derived SF7761 and SF8628 DIPG cells, which both harbour a *H3FRA* gene mutation (H3.3 K27M) were established from biopsies and obtained from Nalin Gupta (University of California, US) [26]. Both cell lines were grown as adherent monolayers. SF7761 cells were maintained in neurobasal A medium (NBA), penicillin streptomycin, B-27 supplement without vitamin A, N-2 supplement, epidermal growth factor (EGF; Peprotech) and fibroblast growth factor (FGF; Peprotech). SF8628 were maintained in Dulbecco’s modified eagle’s medium supplemented with fetal calf serum (FCS). The DUB-D003 cell line was established from a surgical biopsy and carries a *HIST1H3B* gene mutation (K27M H3.1), this cell line was obtained from Chris Jones (Institute of Cancer Research, London, UK) and maintained in NBA supplemented with EGF, FGF, platelet-derived growth factor (PDGF)-AA (Shenandoah Biotech), PDGF-BB (Shenandoah Biotech), B-27 and heparin (StemCell Technologies). Primary rat hippocampal cells were isolated from surgically dissected hippocampi of day 18 embryonic rat brain and maintained in Neurobasal media, pen-strep, L-glutamine, B-27 supplement and FCS. SF7761 and hippocampal cells were maintained as adherent cultures using Laminin (Sigma) or poly-D-lysine (Sigma), respectively. All reagents were purchased from Thermo Fisher Scientific, unless stated otherwise.

All *ex vivo* cell lines were authenticated by short tandem repeat profiling (Public Health England, UK) (S1 Table) and were classified mycoplasma negative (in-house testing).

### Cell viability assay

Cell culture treatments followed an 8-day schedule. Sodium valproate (Sigma) treatment alone was assessed by a 72 hours of continuous drug exposure. Carboplatin (Accord Healthcare Ltd) alone was assessed by two short six hour exposures on two consecutive days followed by removal of media, fresh media added and incubated for an additional 72 hours. Sequential short-term exposures were used to represent the drug infusion time in our clinical CED protocols. Pre-conditioning of cells required exposure to 72 hours of sodium valproate followed by six hours of carboplatin on two consecutive days. Drug was then removed and fresh media containing sodium valproate added for 72 hours.

Assessment of the cytotoxicity of each drug was carried out by MTT (3-(4,5-Dimethylthiazol-2-yl)-2,5-Diphenyltetrazolium Bromide) assay. Briefly, cells were plated at 1000–3000 cells/well in 96-well plates. Following a defined drug incubation period, 10 μl MTT solution was added to each well (5 mg/ml). Cells were then incubated for a further 3–4 hours at 37°C. Cell culture media was aspirated and DMSO:isopropanol was added to solubilise the precipitate. Absorbance was then measured on a FluoSTAR Optima microplate reader (BMG Labtech) at 590 nm, using a reference read at 730 nm. Samples were analysed in triplicate and each experiment repeated a minimum of three times. Values were normalised against the untreated control OD values and calculated as a percentage of control.

### Synergy analysis

Analysis of drug synergy for pre-conditioning experiments was assessed using CompuSyn software following the Chou & Talay method [27]. Fraction affected values were entered for 72 hours sodium valproate alone (drug A); two consecutive six hour doses of carboplatin alone (drug B); and values for pre-treatment with sodium valproate prior to carboplatin exposure (drug A and B in combination). Combination index (CI) values were extrapolated and are indicative of the mode of action for the two drugs. CI values equal to one is indicative of additive effects of both drugs; a CI below one suggests synergy; and a CI above one indicates antagonism between the two drugs.

### Apoptosis assessment

Apoptosis was assessed using the Annexin V-FITC/7-AAD kit (BioLegend), following manufacturer’s instructions. Briefly, cells were plated at 20,000–40,000 cells/well in a 12-well plate, incubated for 16 hours overnight and then dosed with sodium valproate for 72 hours. Cells were then detached from the growing surface and collected. 7-AAD was added to staining buffer at 4 μl per 100 μl of buffer, and FITC-conjugated Annexin V was added at 1 μl per 100 μl buffer. Cells were then diluted 1:1 in staining buffer containing both 7-AAD and Annexin V. Single dyes and unstained cells were used for controls and compensation analysis. All experiments were repeated on at least three occasions.

### Western blotting

Cells were plated at 60,000–100,000 cells/well in a 6-well plate, prior to treating with sodium valproate. Whole cell lysates were obtained from adherent cell cultures by washing cells with ice-cold phosphate buffered saline (PBS) prior to collecting cells in PBS using a cell lifter and added to conditioned media. Cells were then centrifuged at 1500 rpm for five minutes at 4°C. Supernatants were then discarded and pellets washed with PBS followed by a further five minute centrifugation step. PBS was removed and pellets were re-suspended in 50–100 μl of RIPA buffer (Thermo Fisher Scientific) containing protease and phosphatase inhibitors. Cells were incubated on ice for 30 minutes to aid cell lysis. Samples were the centrifuged at 10,500 rpm for five minutes at 4°C to pellet cell particulate. Supernatants were then transferred to fresh tubes and quantified using BCA assay (Pierce, Thermo Fisher Scientific). 10–20 μg of protein were separated by 10% polyacrylamide gel electrophoresis under reducing conditions and transferred to nitrocellulose membrane (Hybond). Membranes were blocked with 5% non-fat dried milk in 1% tris-buffered saline containing 0.1% tween-20 (TBST) for one hour at room temperature prior to probing with acetyl histone H3 antibody (1:5000, Cell Signaling Technology #4353) and rabbit IgG against β-actin (1:50,000, Sigma #A3854) as a protein loading control. To detect apoptotic markers, cleaved caspase 3 (1:1000, Cell Signaling Technology #9664) and cleaved PARP (1:2500, BD Biosciences #556494) antibodies were used. Horseradish peroxidase-labelled secondary anti-rabbit IgG, anti-mouse IgG (Cell Signaling Technology) and enhanced chemi-luminescence (Pierce, Thermo Fisher Scientific) was used to detect immunoreactive bands. Images and densitometry analysis were carried out using Image Lab software (Bio-Rad).

### *In vitro* neurotoxicity assessment by immunofluorescent staining

Immunofluorescent assays were performed on untreated and sodium valproate treated primary hippocampal cells. Experiments were performed on rat E18 hippocampal cultures grown on poly-D-lysine coated glass coverslips after cell extraction had been performed using previously described protocols [28]. Sodium valproate toxicity was assessed at 72 hours post-treatment. B3-tubulin (1:200 Millipore #MAB1637) and GFAP (1:300 Millipore #AB5804) primary antibodies were used to detect the viability of neuronal and glial cells, respectively. Donkey anti-mouse Alexa Fluor488 (Molecular Probes, Thermo Fisher Scientific) and Cy3 (Jackson Labs) were used as secondary antibodies, respectively. The staining protocol required cells to be washed with PBS, fixed using 4% paraformaldehyde and treated with 0.1% triton-X for five minutes to permeabilise cell membranes. Samples were incubated in blocking solution (10% donkey serum) for one hour at room temperature, followed by incubation overnight in primary antibodies at 4°C. Samples were then washed with PBS and incubated with secondary antibodies for one hour at room temperature. The coverslips were then washed three times with PBS and mounted onto microscope slides using 2 μl VectaShield (Vector Labs) containing DAPI nuclear counter-stain. Fluorescently labelled cells were visualised with a Leica DM5500 microscope and digital camera (Leica Microsystems).

### Statistical analyses

For all *in vitro* experiments, data was collected from at least three independent experiments. All statistical analyses were performed on GraphPad Prism^®^ (version 5) with IC_50_ values being derived from logarithmic dose-response curves. The effect of sodium valproate pre-conditioning on carboplatin response was calculated by one-way analysis of variance (ANOVA). Statistical significance was determined using Bonferroni’s multiple comparison post hoc analysis. For comparisons of histone acetylation levels and apoptosis, a one-way repeated measures ANOVA was used with Dunnett’s multiple comparison test. P-values <0.05 were considered significant throughout.

## Results

### Sodium valproate reduces DIPG cell survival *in vitro* and correlates with an increase in histone acetylation

**Fig 1:**
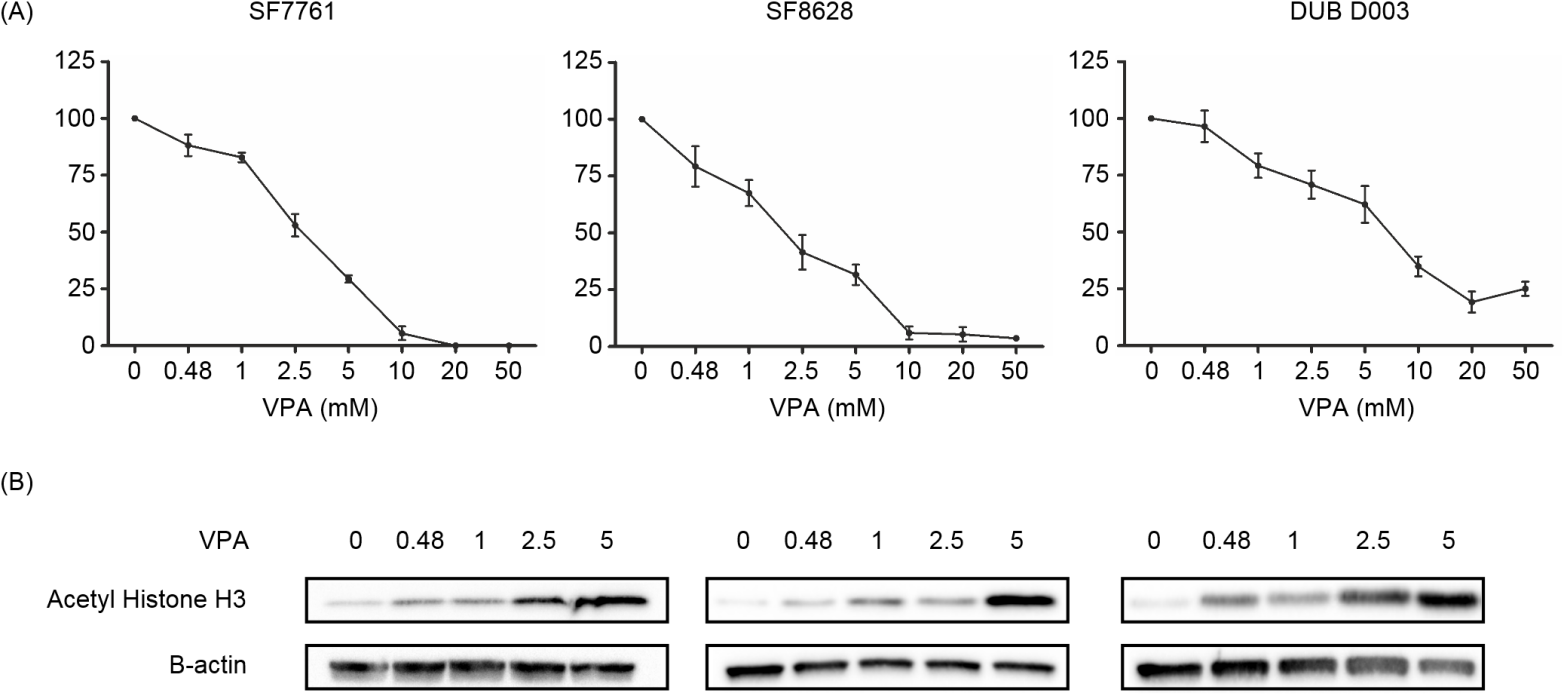
Sodium valproate causes cytotoxicity to DIPG cells and increases histone acetylation. (A) Sodium valproate dose responses for three *ex vivo* DIPG cell lines were conducted using MTT assays, following a 72 hour drug incubation (IC_50_ ranges from 1.8–5.1 mM). (B) Histone acetylation levels were determined by western blotting after 72 hours of sodium valproate treatment and was used as an indicator of HDAC inhibition.

Other HDAC inhibitors (HDACi) have been shown to be cytotoxic towards DIPG cells *in vitro*. This led us to investigate the effect of sodium valproate on the survival of *ex vivo* DIPG cells in culture over a 72 hour period. Sodium valproate had a dose-dependent effect on cell viability (Fig 1A) in all three mutant Histone H3 *ex vivo* cell lines. SF8628 cells were the most sensitive to valproate treatment, with a cytotoxic IC_50_ dose of 1.8 mM, whereas in SF7761 a dose of 2.96 mM was required to achieve an IC_50_ and finally, DUB D003 represented the most resistant and demonstrated an IC_50_ dose over double that of SF8628 at 5.1 mM. Histone acetylation was determined by western blotting, and demonstrated that in all cell lines treated, sodium valproate increased histone H3 acetylation levels in a dose-dependent manner (Fig 1B). At an observational level, as histone H3 acetylation increases, the viability of DIPG cells reduces, suggestive of an inverse correlation between acetylation levels and viability. Western blotting was used to determine the retention of mutant Histone H3 expression in *ex vivo* DIPG cell lines (S1 Fig).

### DIPG cells undergo apoptosis following sodium valproate exposure in a dose-dependent manner

**Fig 2:**
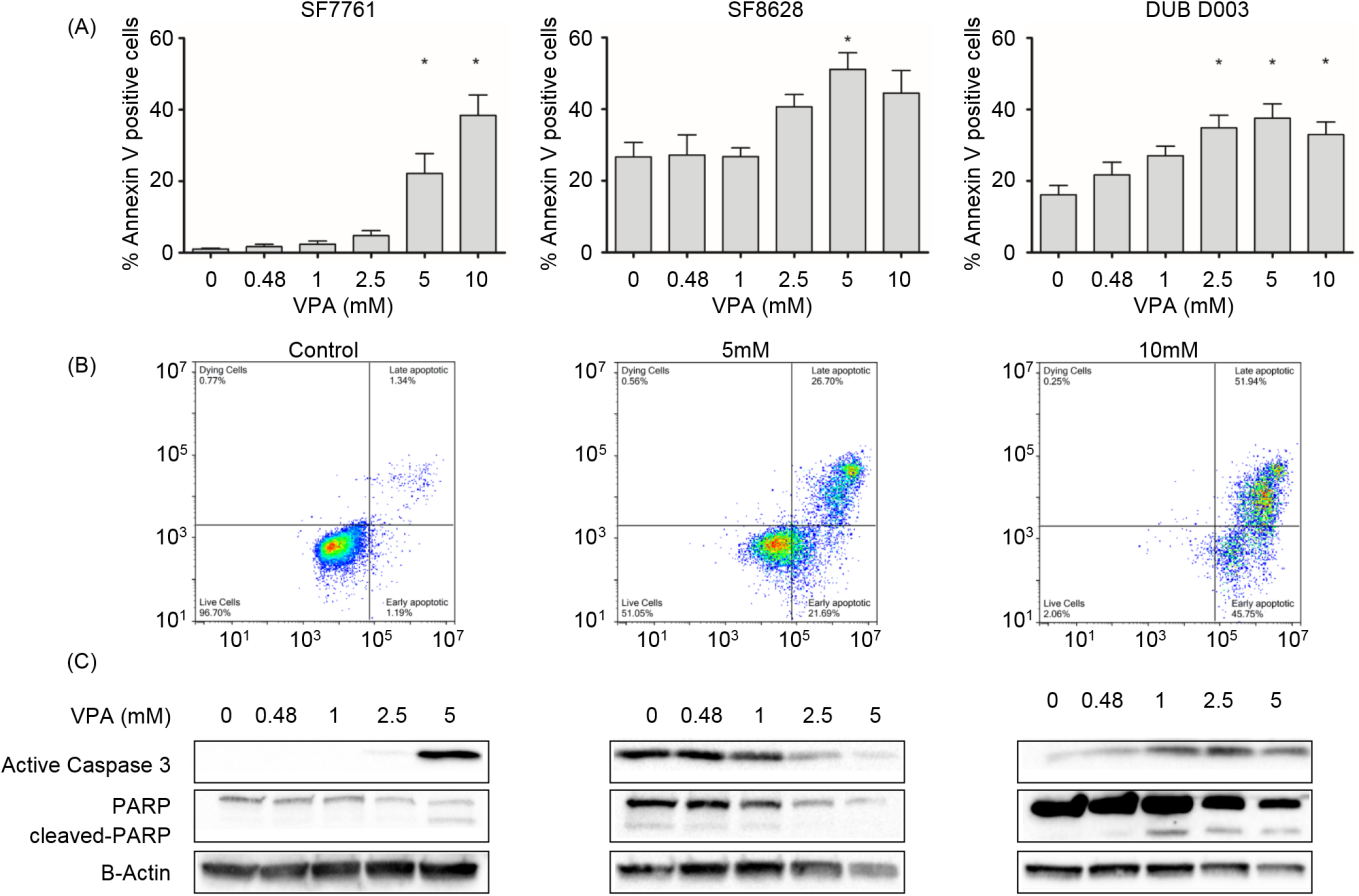
Valproate induced apoptosis in *ex vivo* DIPG cells *in vitro*. Cells were seeded in 12-well tissue culture plates and treated with sodium valproate for 72 hours prior to analysis by flow cytometry for Annexin V and 7-AAD. (A) Dose responses from at least 3 independent experiments were assessed and demonstrate an increase in Annexin V binding occurs from 1–10 mM valproate, with a significant induction of apoptosis occurring at doses of ≥5 mM valproate (p = <0.05). (B) Representative images of apoptosis analysis, all experiments were compensated based on single dyes alone. (C) To confirm the induction of apoptosis western blotting was carried out on all three cell lines treated with ≤5 mM valproate for 72 hours. * denotes statistical significance p = <0.05.

To further understand how sodium valproate reduces cell viability, we evaluated the induction of apoptosis by Annexin V and 7-AAD staining utilising flow cytometry. Analysis of cell populations treated with sodium valproate for 72 hours, demonstrated a significant induction in apoptosis when DIPG cells were treated with 5 mM sodium valproate (p = <0.05; Fig 2A) in all three DIPG cell lines tested. It is interesting to note, that both SF8628 and DUB D003 both show some positive Annexin V staining in untreated cells, whereas minimal positivity was apparent in SF7761. The apoptotic markers, cleaved-PARP and cleaved caspase 3 were evident in SF7761 when treated with 5mM sodium valproate, confirming the results of the flow cytometric analysis of Annexin V staining (Fig 2B), these results corroborate findings from cell viability assays. Similar findings were demonstrated in DUB D003 cells. With an increase in cleaved PARP and cleaved caspase 3 in a dose dependent manner. However, SF8628, showed a decrease in both cleaved PARP and caspase 3. Like histone acetylation levels and cell viability, apoptosis, indicated by Annexin V positivity, occurs in a dose-dependent manner in all three cell lines. Treatments above 10 mM of sodium valproate demonstrated strong cytotoxic effects against DIPG cells, with a higher proportion of late apoptotic/necrotic cells.

### Sodium valproate causes minimal toxicity to normal rat hippocampal cells

**Fig 3:**
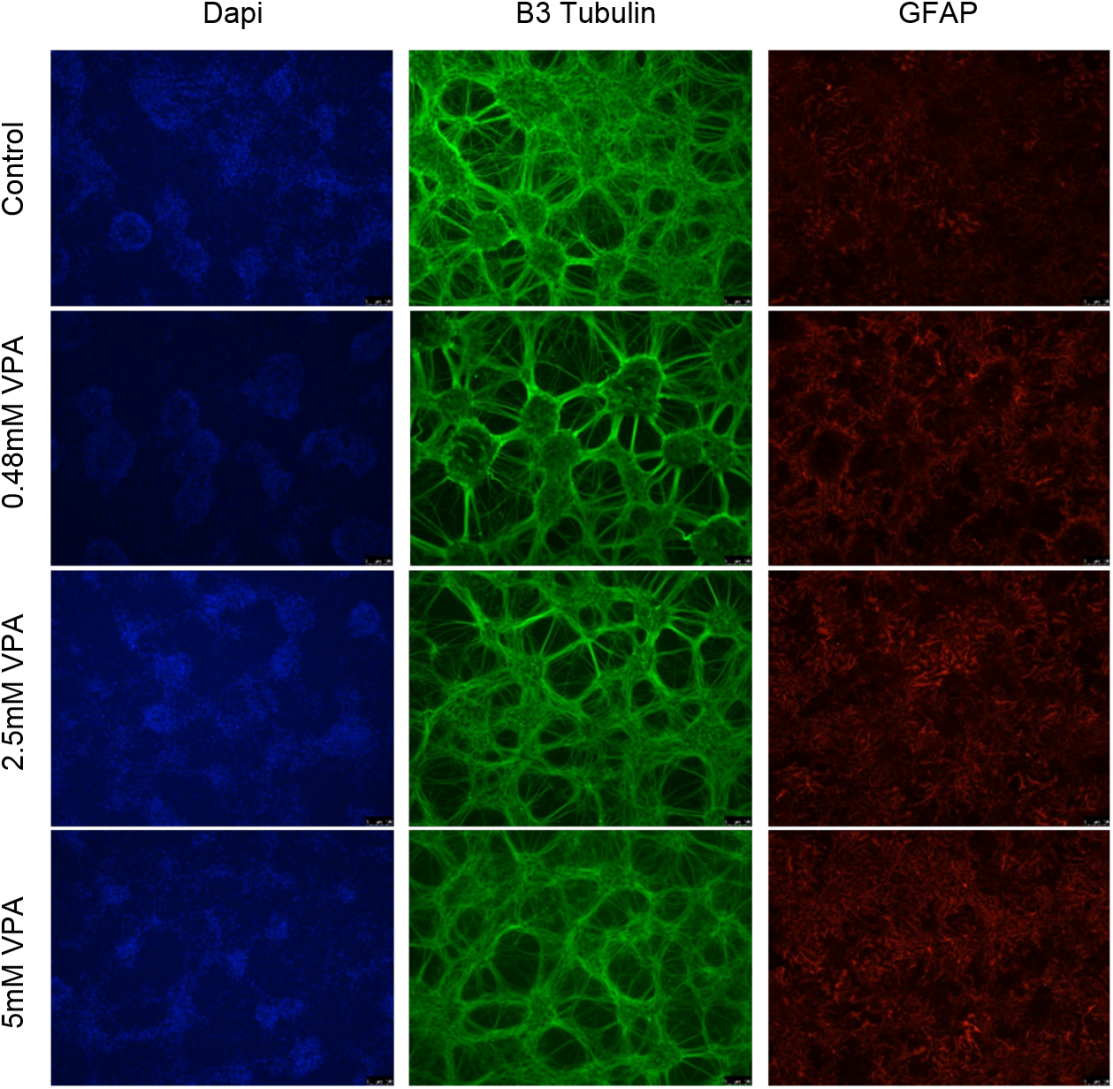
*In vitro* primary neuronal and glial cell toxicity analysis. Primary rat hippocampal cells were dosed with valproate for 72 hours and assessed by immunofluorescent staining of neuronal (B3 tubulin) and glial (GFAP) markers. Hippocampal cultures show intact neuronal networks at 5 mM valproate and glial cells with normal morphology indicative of no significant toxicity.

We and others have shown sodium valproate to cause cytotoxicity to cancer cells; we used normal hippocampal cells obtained from rat hippocampi as mixed glial and neuronal cultures to study the cytotoxic effects on normal brain cells. Sodium valproate treatment was associated with minimal toxicity to both neuronal and glial cells (Fig 3). Neuronal cells identified by B3 tubulin (green) positive staining demonstrates intact neuronal networks. Furthermore, glial cells marked by GFAP (red) staining show normal morphology.

### Short term carboplatin exposure mimicking clinical CED infusions causes cytotoxicity to *ex vivo* DIPG cells

**Fig 4:**
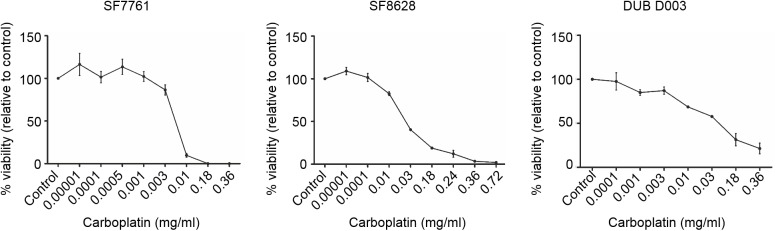
Anti-tumour effect of repeated 6h carboplatin exposures to DIPG cells. To mimic convection enhanced delivery infusion times, DIPG cells were treated for two six hour periods with carboplatin followed by assessment of cell viability 72h later. DUB D003 is the most resistant to short term carboplatin exposure with an IC_50_ of 0.047mg/ml, followed by SF8628 with an IC_50_ of 0.026 mg/ml whilst SF7761 has an IC_50_ of 0.0048 mg/ml.

Next, owing to the increased histone acetylation levels seen with sodium valproate, we proposed to determine whether or not this drug could enhance cytotoxic effects of carboplatin on DIPG cells, a drug currently used for the treatment of high grade gliomas by CED. CED utilises an intraparenchymal catheter system to repeatedly infuse drugs directly into the brain. Cell viability assays demonstrated that two six hour exposures to carboplatin caused cytotoxic effects visible at 72 hours post treatment. Dose responses demonstrate that DUB D003 is the most carboplatin resistant cell line with an IC_50_ of 0.047 mg/ml, whereas SF8628 has an IC_50_ of 0.026 mg/ml. SF7761 is most sensitive cell line to carboplatin with an IC_50_ dose of 0.0048 mg/ml, nine-fold lower than that of DUB D003 (Fig 4).

### Valproate pre-conditioning of DIPG cells potentiates the cytotoxic effects of carboplatin *in vitro*

Finally, an *in vitro* pre-treatment strategy was devised using 0.48 mM, 1 mM and 2.5 mM sodium valproate for SF8628, DUB D003 and SF7761 cells, respectively, owing to the insignificant effect on cell apoptosis seen in each cell line at the given dose.

**Fig 5:**
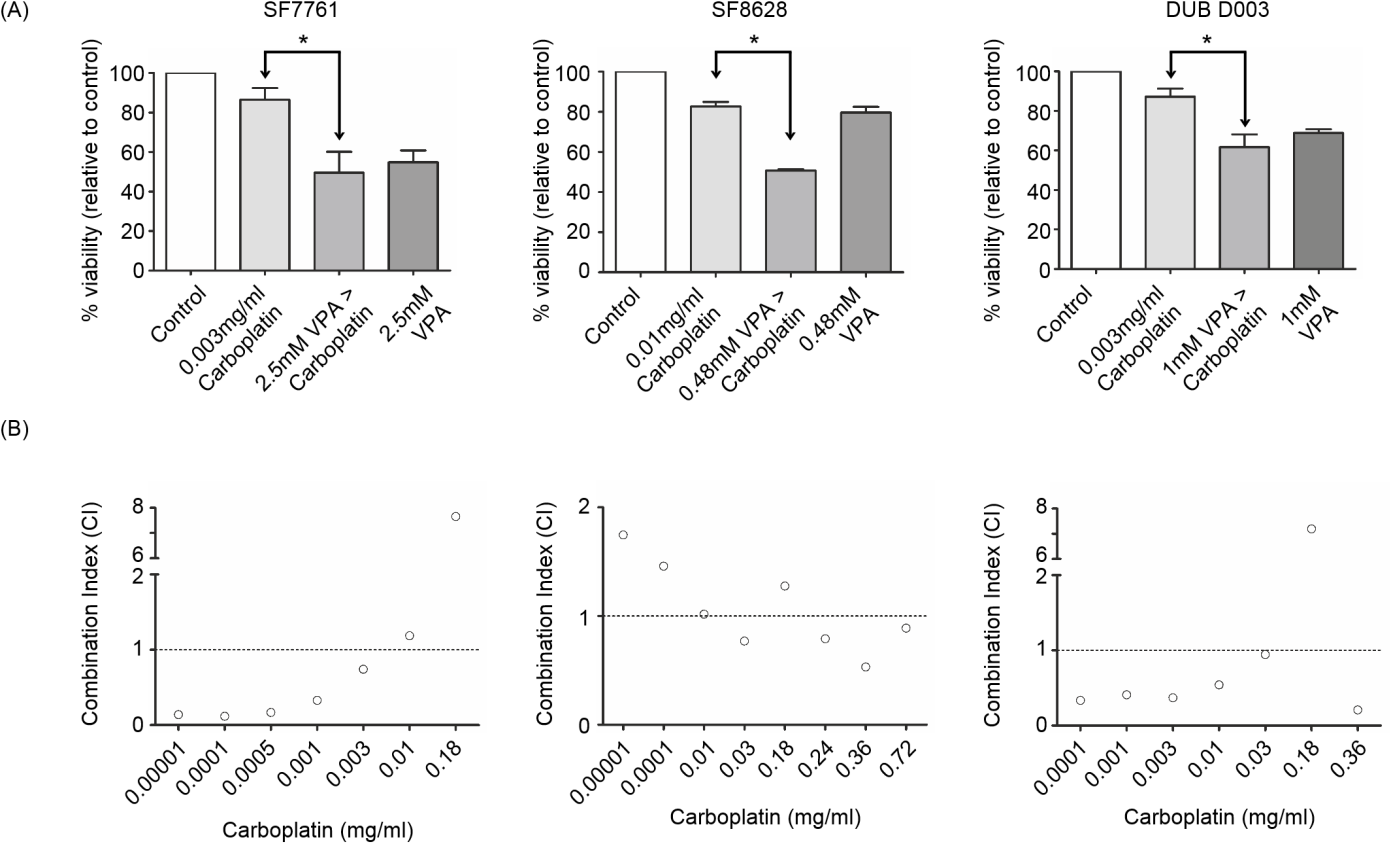
Anti-tumour effect of carboplatin on DIPG cells pre-treated with sodium valproate. DIPG cells were treated with valproate for 72 hours followed by two six hours carboplatin treatments and a final 72 hours of valproate. (A) Pre-treatment of SF7761, SF8628 and DUB D003 with sodium valproate followed by carboplatin, bars represent mean of at least three independent experiments, error bars indicate standard error. * p<0.05, assess by ANOVA. (B) To determine synergy, combination index (CI) values were calculated by CompuSyn software, and plotted against carboplatin dose using GraphPad Prism software. CI values below 1 (dotted line through y-axis), represent synergistic combinations.

Pre-treatment of DIPG cell cultures with sodium valproate prior to carboplatin, potentiates the cytotoxic effect of carboplatin significantly in comparison to carboplatin alone in three DIPG cell lines (p = <0.05; Fig 5A). Interestingly, the treatment of SF8628 with sodium valproate followed by carboplatin resulted in a significant difference (p = 0.05) compared to both carboplatin alone and sodium valproate alone. In comparison, pre-treatment of SF7761 and DUB D003 resulted in a significant reduction in cell viability compared to carboplatin alone, but only a slight decrease was seen in comparison to sodium valproate alone.

Synergy analysis of combined drug treatments demonstrated that the augmentation of carboplatin cytotoxicity by sodium valproate pre-treatment is synergistic, when compared to carboplatin alone. SF8628 cells in general, show synergy when combining sodium valproate with higher doses of carboplatin (0.03–0.72 mg/ml), whereas the synergistic effects of sodium valproate and carboplatin are exerted at lower doses of 0.0001–0.003 mg/ml carboplatin for SF7761 and 0.0001–0.03 mg/ml in DUB D003 cells (Fig 5B).

## Discussion

Repurposing sodium valproate as an adjuvant therapy for DIPG carries many advantages over other novel agents; namely its well characterised toxicity and pharmacokinetics profile. Here, we have demonstrated the *in vitro* efficacy of sodium valproate as a cancer therapeutic and its ability to enhance the cytotoxic effects of carboplatin on mutant Histone H3 DIPG cells *in vitro*.

Anti-cancer activity of sodium valproate occurs in all three human DIPG cell lines tested *in vitro*, with the IC_50_ ranging from 1.8–5.1 mM following a 72 hour drug exposure. These findings are consistent with other *in vitro* cancer studies, which have demonstrated IC_50_ ranging from 3–10 mM for ovarian and uterine sarcoma cells, and 4.5–8 mM for thoracic (oesophageal and non-small cell lung) cancer cells [29, 30]. Much lower cytotoxic IC_50_ of 0.89–1.92 mM have been noted in neuroendocrine tumours, but this required drug exposure times of up to seven days [31].

The cell lines tested in this study all harboured histone H3 K27M mutations. Recent reports have demonstrated no significant difference in the sensitivity of wild-type and mutant histone H3 DIPG cells [16, 32]. These studies have used panobinostat, another HDAC inhibitor, which is currently in clinical trials for DIPG treatment, and showed cell toxicity to occur independently of histone H3 mutation status [16, 32].

We have confirmed that sodium valproate causes HDAC inhibition in DIPG cells, by demonstrating increased histone H3 acetylation levels in a dose-dependent manner. Two studies have suggested that HDAC inhibition in bladder cancer and colon cancer cells, causes an induction in p21 expression, which results in the inhibition of cell growth and apoptosis [33, 34]. Further investigation is required to determine whether HDAC inhibition by sodium valproate leads to p21 induction and cell cycle arrest. We have shown an inverse correlation between histone H3 acetylation and cell viability, however, we have not yet proven that HDAC inhibition is the direct mechanism for the anti-cancer activity of sodium valproate. Interestingly, Grasso and colleagues demonstrated that a dose-dependent increase in histone H3 acetylation and increased H3K27-trimethylation following panobinostat treatment gave rise to a partial rescue of the H3K27M-induced global hypotrimethylation phenotype [16]. Whilst differences exist between the targets of panobinostat and valproate, it would be interesting to establish if these detoxifying effects can also be achieved with valproate treatment, or are purely a result of pan-histone acetylation with panobinostat.

We aimed to determine the mechanism of cell death induced by sodium valproate and as such pursued further investigation into phosphatidylserine (PS) externalisation in DIPG cells, which is an indicator of cells undergoing apoptosis. The externalisation of PS can be detected by Annexin V binding. In all three DIPG cells, there was an increase in Annexin V binding in sodium valproate treated cells, in a dose-dependent manner. Apoptosis was further confirmed in two out of three cell lines by an induction of cleaved PARP and caspase 3. One cell line, SF8628, showed the opposite effect on these apoptotic markers. One reason for this may be due to 20% of cells labelling positive for Annexin V in untreated cells, and subsequent treatment with sodium valproate pushes a higher proportion of cells through to late apoptosis and/or primary necrosis. Alternatively, Annexin V staining could be a result of primary necrosis and not apoptosis, as one report demonstrates that monocytic leukaemia cells that undergo primary necrosis, stain positive for Annexin V prior to staining with propidium iodide [35].

HDACs are responsible for the removal of acetyl groups from core nucleosome histones and as such, result in chromatin compaction and transcriptional repression. This reduction in transcriptional activity could result in the silencing of pro-apoptotic genes, such as *BAX* and *BAK*. Indeed, in the breast cancer cell line MCF7, treatment with valproic acid resulted in apoptosis, which correlated with a down-regulation of the anti-apoptotic protein Bcl-2 and up-regulation of Bak. Furthermore, treatment of chronic lymphocytic leukemia cells led to a reduction in the Bcl-2/Bax ratio [36, 37].

HDAC inhibition is postulated to alter chromatin structure and as such, may allow DNA intercalating agents such as carboplatin, greater access to the DNA. Owing to this, we have studied the combined use of sodium valproate and carboplatin for the treatment of DIPG. Our findings demonstrated that short-term sequential treatment of cells *in vitro* with carboplatin causes significant cytotoxicity to DIPG cells. Collectively, we have also shown that pre-conditioning of cells with sodium valproate prior to carboplatin dosing potentiates this cytotoxicity. As an anti-seizure medication, it is not uncommon for brain tumour patients to be treated with sodium valproate. A retrospective study of DIPG patients has shown that those given sodium valproate in addition to standard therapy (radiotherapy, carboplatin and vincristine), demonstrated 13.4 months overall survival compared to 7.8 months for those without valproate treatment [38], whilst in glioblastoma, the addition of valproate treatment was associated with a 28% decrease in risk of death [39]. Furthermore, studies have demonstrated other HDACi such as vorinostat can potentiate effects of carboplatin and paclitaxel [40], increasing the response rate in patients with advanced solid malignancies to 53%, compared to 20–30% for carboplatin-paclitaxel alone. Our observations have confirmed our hypothesis that sodium valproate potentiates the cytotoxic effects of carboplatin on DIPG cells *in vitro*. Although, the mechanism responsible for the synergy between these two drugs has not yet been determined, one could surmise that HDAC inhibition by sodium valproate results in chromatin remodelling, thereby relaxing the chromatin structure and allowing more carboplatin-DNA adducts to form. Supporting this conjecture, HeLa cells treated with as little as 0.05 mM valproate for 1 hour, showed a 48% decrease in condensed chromatin area, which was sustained over 24 hours with a 32% decrease at the longest time point [41]. Whilst breast cancer cells treated with 2 mM sodium valproate demonstrated a down-regulation of structural maintenance of chromatin protein 1 (SMC1), DNA methyltransferase 1 (DNMT1) and heterochromatin protein 1 (HP1), proteins known to maintain heterochromatin structures [42].

This study demonstrates the potential for sodium valproate to be used as a monotherapy and as a sensitising agent for carboplatin. The synergy seen when sodium valproate is combined with carboplatin, highlights the potential of sodium valproate to be used in combination with many other drugs including temozolomide for treatment of glioblastoma. Interestingly, studies have previously shown that valproic acid can re-sensitise cells to previously used cytotoxic chemotherapies, suggesting it could be used when a tumour has become resistant to a particular therapy [43].

Extrapolated data from dose response experiments determined that at the highest clinically achievable level of sodium valproate, specifically 1.5 mM [44], sodium valproate causes an average of 20% reduction in viability across the three cell lines. Given that *in vitro* the average IC_50_ dose across all DIPG cell lines tested is 2.8 mM, and assuming that only 15% of the clinical serum level reaches the brain [45], it would seem unlikely to achieve therapeutic doses at the tumour site through systemic drug administration without first incurring significant toxicity. This advocates the need for other delivery methods to be used to achieve cytotoxic concentrations of sodium valproate at the tumour site, if it were to be used as a monotherapy. Being a water soluble drug, sodium valproate is a potential candidate for delivery via CED. We are currently undertaking preclinical work to ascertain its suitability for delivery by CED for the treatment of malignant glioma.

In summary, we demonstrate *in vitro* anti-glioma activity of sodium valproate in mutant histone H3 DIPG cells. Sodium valproate caused a reduction in cell viability, mainly through the induction of apoptosis and an increase in histone H3 hyperacetylation, which is indicative of HDAC inhibition. The cytotoxic effects of short-term carboplatin treatment *in vitro*, can be potentiated by pre-conditioning DIPG cells with sodium valproate. Whilst the exact mechanism is not known for the synergistic effects of these two drugs, this data clearly advocates further study to test the efficacy in a relevant *in vivo* DIPG model and supports the use of sodium valproate as an adjuvant chemotherapeutic.

## Supporting information

S1 Fig Confirmation of K27M mutant Histone H3 expression in *ex vivo* DIPG cells. Western blotting was used to confirm if the *ex vivo* DIPG cells used in the study retained mutant Histone H3 expression. The anti-Histone H3 (K27M mutant) antibody (Millipore, ABE419) was used at a concentration of 1:1000 followed by 1:2500 dilution of secondary anti-rabbit.
(TIF)

S1 Table Short tandem repeat (STR) profiling of *ex vivo* DIPG cell lines. (DOCX)
