# Supplementary figures and images for: Repurposing the anti-epileptic drug sodium valproate as an adjuvant treatment for diffuse intrinsic pontine glioma
Source: PLoS One. 2017 May 25;12(5):e0176855. doi: 10.1371/journal.pone.0176855 (PMC5444593; doi:10.1371/journal.pone.0176855)

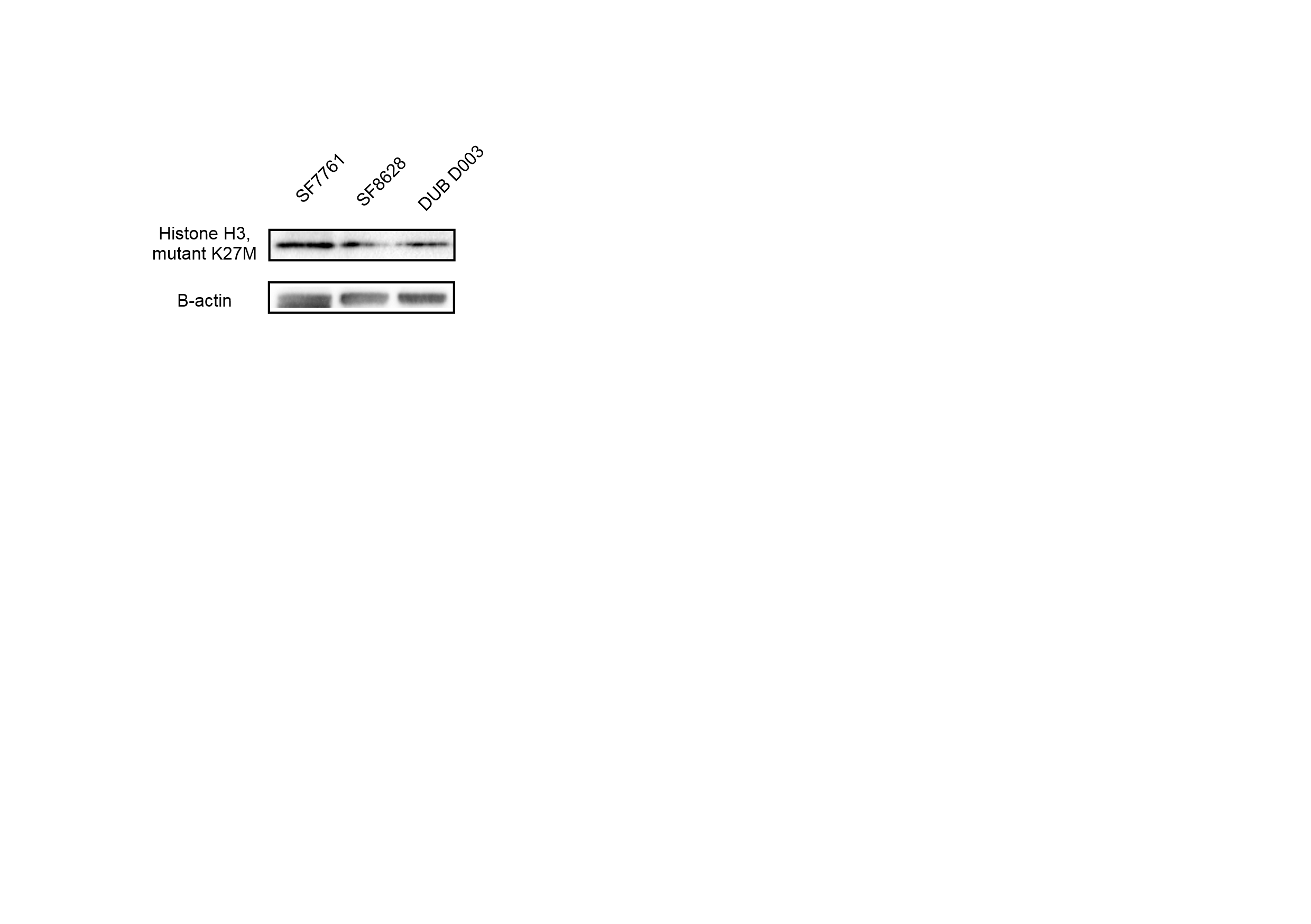

Supplement: S1 Fig — Western blotting was used to confirm if the ex vivo DIPG cells used in the study retained mutant Histone H3 expression. The anti-Histone H3 (K27M mutant) antibody (Millipore, ABE419) was used at a concentration of 1:1000 followed by 1:2500 dilution of secondary anti-rabbit. (TIF) [file pone.0176855.s001.tif]
