# Supplementary material for: Repurposing the anti-epileptic drug sodium valproate as an adjuvant treatment for diffuse intrinsic pontine glioma
Source: PLoS One. 2017 May 25;12(5):e0176855. doi: 10.1371/journal.pone.0176855 (PMC5444593; doi:10.1371/journal.pone.0176855)
Supplement: S1 Table — (DOCX) [file pone.0176855.s002.docx]

S1 Table.

|  | DUB D003 | SF7761 | SF8628 |
| --- | --- | --- | --- |
| D3S1358 | 16, 18 | 15, 17 | 15, 18 |
| THO1 | 9, 9.3 | 7, 9.3 | 7, 7 |
| D21S11 | 31, 31.2 | 28, 31.2 | 29, 30 |
| D18S51 | 14, 14 | 19, 22 | 14, 14 |
| PENTA E | 11, 16 | 14, 17 | 11, 13 |
| D5S818 | 12, 13 | 11, 12 | 11, 12 |
| D13S317 | 11, 11 | 8, 9 | 9, 9 |
| D7S820 | 11, 12 | 10, 11 | 11, 11 |
| D16S539 | 9, 9 | 11, 11 | 9, 9 |
| CSF1PO | 10, 11 | 13, 13 | 11, 12 |
| PENTA D | 13, 13 | 13, 13 | 11, 12 |
| AMEL | X, X | X, X | X, x |
| vWA | 16, 19 | 14, 18 | 16, 17 |
| D8S1179 | 13, 15 | 11, 15 | 10, 13 |
| TPOX | 8, 11 | 11, 12 | 8, 8 |
| FGA | 22, 25 | 20, 20 | 22, 22 |
